# Comparison of e-cigarette use prevalence and frequency by smoking status among youth in the United States, 2014–19

**DOI:** 10.1111/add.15439

**Published:** 2021-02-21

**Authors:** Jamie Tam, Andrew F. Brouwer

**Affiliations:** 1 Yale University School of Public Health, New Haven, 60 College St, New Haven, CT, 06510, USA; 2 and Department of Epidemiology, University of Michigan, Ann Arbor, MI, USA

**Keywords:** Cigarette, smoking, adolescent smoking, cross-sectional survey, e-cigarettes, electronic cigarettes, smoking, United States, youth

## Abstract

**Background and aims:**

Reports of youth e-cigarette use often do not disaggregate by underlying smoking status. This study compared annual 2014–19 youth estimates of past 30-day e-cigarette use prevalence and frequency by smoking status in the United States.

**Design:**

Nationally representative, cross-sectional, school-based survey [National Youth Tobacco Surveys (NYTS)]. General linear models accounting for complex survey design compared e-cigarette use prevalence by smoking status by year, overall and stratified by frequency, separately for high school (HS) and middle school (MS) students. The 2019 survey was analyzed separately because of its change in survey methodology.

**Setting:**

MSs and HSs in the United States.

**Participants:**

A total of 116 704 students from 1268 schools, ages 9–19.

**Measurements:**

Students self-reported (paper 2014–18, electronic 2019) ever and past 30-day (current) use of e-cigarettes and cigarettes, as well as frequent use (20–30 days of month).

**Findings:**

From 2014 to 2018, current e-cigarette use prevalence increased among never, current and former smokers in HS, but only among never and current smokers in MS (each *P*-value < 0.001). E-cigarette use increases for current HS smokers were primarily among frequent e-cigarette users. In 2018, the absolute number of HS frequent users who were never or former smokers (420 000 combined) surpassed current smokers (370 000). In 2019, current e-cigarette use prevalence for never, former and current smokers was 17.5% [95% confidence interval (CI) = 16.0–19.0], 53.6% (95% CI = 45.2–61.9) and 85.8% (95% CI = 81.6–89.9) for HS students, respectively, and 6.8% (95% CI = 5.9–7.7), 40.8% (95% CI = 34.7–47.0) and 78.0% (95% CI = 71.9–84.2) for MS students. That year, the number of HS never (420 000) and former smokers (570 000) using e-cigarettes frequently eclipsed that of current smokers (390 000).

**Conclusions:**

E-cigarette use prevalence and frequency among youth vary by smoking status, with highest levels of use among current smokers. However frequent e-cigarette use among never smokers and former smokers has increased.

## INTRODUCTION

Surveys show unprecedented increases in e-cigarette use among United States youth since 2017 [1–4]. High school students’ e-cigarette use increased in 2018, with 20.8% of high school students reporting use of an e-cigarette within the past month [2]. In 2019, that increased to 27.5% [4]; however, patterns of e-cigarette use among youth differ dramatically by smoking status, with much higher rates of use among current smokers compared to never smokers [5–8]. E-cigarette use is more common among youth who have already used other tobacco products [9]. For young people who smoke cigarettes and use e-cigarettes, their cigarette use presents the greater health risk. However, e-cigarettes present new health risks for young people who have never smoked or used tobacco products, because 99% of them contain nicotine [10]. Data from the 2018 National Youth Tobacco Survey (NYTS) show that use of e-cigarettes rose among youth who were naive to other tobacco products [11]. However, the 2018 NYTS data probably underestimated e-cigarette use due to the exclusion of JUUL from the list of examples for e-cigarettes—only in 2019 did the survey begin to explicitly mention ‘JUUL’ as a type of e-cigarette [4].

Public health leaders are especially concerned that never smoker youth might become addicted to nicotine through e-cigarettes, and that flavors could be the ‘on-ramp’ to addiction [12]. In response to rising e-cigarette use among youth and an outbreak of e-cigarette lung injuries [primarily attributed to off-market tetrahydrocannabinol (THC) vaping liquid] [13], several states enacted emergency rules to temporarily restrict sales of flavored e-cigarettes, and the Food and Drug Administration (FDA) removed certain flavored cartridge-based e-cigarettes, such as JUUL [14,15].

For never smoking youth who use e-cigarettes, the extent to which they are at risk for nicotine addiction, in theory, depends on their frequency of exposure. Research from 2014 to 2015 showed that high e-cigarette use frequency was concentrated predominately among youth who already use combustible tobacco products, with very low levels of frequent e-cigarette use among never smokers [7,9,16]. Data from the 2014 Monitoring the Future survey also showed that, among non-smoking high school students who use e-cigarettes, most used them on 1–2 of the past 30 days [3]. Frequent use of e-cigarettes is associated with nicotine dependence among youth [17], although never smokers who are using e-cigarette infrequently may not be addicted to nicotine. An analysis of data from the 2015–17 NYTS examined frequency of e-cigarette use among all students, but did not disaggregate results by smoking status [18]. If e-cigarette use frequency has been increasing among never smokers over time, that could present cause for concern.

In this study, we compare past 30-day use of e-cigarettes for high school (HS) and middle school (MS) students in the 2014–19 NYTS surveys by year and smoking status (never, current, former), overall and stratified by frequency of e-cigarette use. We considered HS and MS students separately because they represent distinct age groups, cultures and, typically, physical locations and have different tobacco use patterns.

## METHODS

### Design and participants

The NYTS data offer timely information on trends in tobacco product use among youth and can monitor changes in both frequent, current and ever use of e-cigarettes. The NYTS is a nationally representative, school-based cross-sectional survey conducted among MS (grades 6–8; primarily aged 11–13 years) and HS (grades 9–12; primarily aged 14–18 years) students that collects information about tobacco-related behaviors. It uses a stratified, three-stage cluster sampling procedure accounting for primary sampling units, schools and students [19–21]. It is a voluntary, self-administered, pencil-and-paper (2014–18) survey conducted in public and private schools with response rates (sample sizes; number of participating schools) as follows: 2014: 73.3% (22 007; 207); 2015: 63.4% (17 711; 185); 2016: 71.6% (20 675; 202); 2017: 68.1% (17 872; 185); 2018: 68.2% (20 189; 238); and 2019: 66.3% (19 018; 251). Unlike previous survey years, the 2019 NYTS was conducted using electronic data collection for the first time instead of paper-and-pencil questionnaires. Additional details can be found on-line [19].

### Measures

Beginning in 2014, the NYTS measured frequency of e-cigarette use with the question: ‘During the past 30 days, on how many days did you use electronic cigarettes or e-cigarettes such as Blu, 21st Century Smoke, or NJOY?’, with seven possible response options: 0 days, 1–2 days, 3–5 days, 6–9 days, 10–19 days, 20–29 days and all 30 days. In 2015, language describing specific brands ‘such as Blu, 21st Century Smoke, or NJOY’ was removed from the question, and then from 2016 to 2019, the question was revised to simply ask: ‘During the past 30 days, on how many days did you use e-cigarettes?’. NYTS data from 2014 to 2018 may potentially underestimate e-cigarette use among youth, because the NYTS did not make explicit mention of the JUUL brand in its questions about e-cigarette use until 2019. The 2019 NYTS added the brand example ‘JUUL’ in 2019 by prompting students with: ‘The next several questions are about electronic cigarettes or e-cigarettes. Some brand examples include JUUL, Vuse, MarkTen, and blu.’ Among those who reported any e-cigarette use in the past 30 days, three frequency use categories are considered: infrequent (1–5 days), moderate (6–19 days) and frequent (20–30 days). Because of low numbers, we collapsed moderate and frequent use among MS students. Current use of e-cigarettes is defined as any use within the past 30 days.

Smoking status was assessed with the question: ‘Have you ever tried cigarette smoking, even one or two puffs?’. ‘Never smokers’ are those who responded ‘no’. Youth who have smoked cigarettes at all within the past 30 days are considered to be ‘current smokers’. ‘Former smokers’ are those who responded ‘yes’, but who have not smoked at all within the past 30 days. Distributions of e-cigarette use frequency among never, former and current smokers for MS and HS students were calculated. We focus on cigarette smoking because it is the leading cause of preventable death [22], and therefore the target of most tobacco prevention efforts. This also allows us to provide results that can be compared with previous reports [5,7]. However e-cigarette users may also be co-using with other combustible tobacco products, such as cigars, pipe tobacco, hookah or bidis. For parallel results with respect to all combustible tobacco products, see the Supporting information.

### Analyses

We calculated the weighted prevalence of past 30-day e-cigarette use in each year for MS and HS students of each smoking status, overall and by e-cigarette use frequency. Using general linear models with a logit link function and accounting for complex survey design, we estimated the effects of year alone and then smoking status, year and interactions between smoking status and year on past 30-day e-cigarette use prevalence, overall and stratified by e-cigarette use frequency. Models were not adjusted to eliminate non-significant interaction terms. We developed these models for MS and HS students separately. Each of these models simultaneously test for differences between smoking status and across survey years. All analyses account for the complex survey design of NYTS by incorporating the provided primary sampling units (PSU), strata and weights using the ‘survey’ package in R version 4.0. Statistical testing used the ‘car’ package [23–25]. *P*-values given in the text and tables represent tests of the specific comparisons indicated rather than of main effects or interaction effects *per se*. The NYTS 2019 data are excluded from the statistical models, because the change in the NYTS format from paper to electronic beginning in 2019 prohibits direct statistical comparison with previous years.

Consistent with national reports [3], results were suppressed when the unweighted denominator was < 50 or when the relative standard error was *>* 30% to exclude estimates that could be statistically unreliable. Those with missing data for e-cigarette use and cigarette smoking were treated as missing observations. Because each sample is nationally representative, we estimated the total number of students that each weighted prevalence corresponds to nationally, as specified by the survey, rounded-down to the nearest 10 000 people. This analysis was not pre-registered, and the results should be considered exploratory.

## RESULTS

Table 1 describes the sample (116 704 students from 1268 schools), including sex, age (9–19), grade, race/ethnicity, smoking status and e-cigarette use status, stratified by year and HS versus MS. Missing data on smoking status accounted for ≤ 3% of samples; for e-cigarette use status they accounted for ≤ 2.0%.

Prevalence by year and smoking status is given for both HS and MS students in Table 2. Among all students from 2014 to 2018, past 30-day use of e-cigarettes increased (*P*-value < 0.001) among HS students from 13.4 to 20.8%, but did not significantly change (*P*-value = 0.11) for MS students (3.9% in 2014 and 4.9% in 2018). Although the data are not directly comparable with preceding years, in 2019 more than a quarter of HS students and more than one in 10 MS students used e-cigarettes in the past month.

From 2014 to 2018, past 30-day e-cigarette use prevalence varied significantly by smoking status—with the highest prevalence among current smokers and the lowest among never smokers—for both HS students (*P*-value for never versus current user: < 0.001; for never versu*s* former user: < 0.001; current versu*s* former user: < 0.001) and MS students (*P*-value for never versu*s* current user: < 0.001; for never versu*s* former user: < 0.001; current versu*s* former user: < 0.001). Prevalence increased between 2014 and 2018 for HS students of all smoking statuses: 4.7–11.7% for never smokers, 23.0–38.9% for former smokers and 56.6–71.0% for current smokers. The largest jump in prevalence for each group occurred between 2017 and 2018. Although not directly comparable with preceding years, 2019 estimates show that 17.5% of never smokers, 53.6% of former smokers and 85.8% of current smokers in HS used e-cigarettes in the past month.

For MS students, 30-day e-cigarette use prevalence was lower than for HS students overall. Prevalence increased between 2014 and 2018 for MS never smokers (1.4–2.8%) and current smokers (45.8–72.8%), but not for former smokers (14.6–14.6%). Again, the greatest single-year increase for never and current MS smokers was 2017–18. Data from 2019 show that among MS students, 6.8% of never smokers, 40.8% of former smokers and 78.0% of current smokers used e-cigarettes in the past month.

In Tables 3 (HS) and 4 (MS), we stratify prevalence by frequency of e-cigarette use. Between 2014 and 2018, prevalence of use increased significantly for never smokers in each frequency group (each *P*-value < 0.001). For former smokers, the increases were significant for moderate and frequent use only (*P*-values = 0.003, < 0.001). When testing for year-to-year increases, significant changes occurred in infrequent, moderate and frequent e-cigarette use from 2017 to 2018 among never and former smokers. For current smokers, only prevalence of frequent use increased significantly (*P*-value < 0.001) and, again, the year-to-year increase was significant from 2017 to 2018 (15.2–32.7%); no other years had a significant difference. Although the prevalence of frequent e-cigarette use among HS student has been highest among current smokers, the estimated absolute number of frequent e-cigarette users who are never (170 000) or former (250 000) smokers together surpassed the number of frequent e-cigarette users who are current smokers (370 000) in 2018. The 2019 data show that among HS students, while the prevalence of frequent use remains lower among never and former smokers (3.7 and 23.2%) compared to current smokers (46.1%), the absolute number of never smokers (420 000) and former smokers (570 000) using e-cigarettes frequently exceeds that of current smokers (390 000).

For MS students (Table 4), prevalence of both infrequent and moderate–frequent use increased significantly between 2014 and 2018 for never smokers (*P*-values = 0.004, < 0.001), while no significant increase was observed for any frequency category for former smokers. Moderate-to-frequent use increased for current smokers (*P*-value = 0.004). By 2018, 0.6% of never smokers, 5.7% of former smokers and 43.4% of current smokers were moderate–frequent e-cigarette users. The 2019 data show that 1.8% of never smokers (180 000 MS students), 17.9% of former smokers (120 000) and 41.7% of current smokers (110 000) used e-cigarettes on a moderate–frequent basis.

Findings by combustible tobacco use status are similar to those by smoking status: past 30-day and frequent e-cigarette use prevalence is highest among current combustible tobacco users, followed by former combustible tobacco users, and lowest among those who have never used combustible tobacco (Supporting information, Tables S1–S3).

## DISCUSSION

In 2019, it was reported that current e-cigarette use increased to 27.5% among HS students and 10.5% among MS students [4], but these estimates mask large differences by underlying smoking status. During that year, we find the prevalence of e-cigarette use was 17.5, 53.6 and 85.8% for never, former and current smokers in HS, respectively, and 6.8, 40.8 and 78.0% for never, former and current smokers in MS, respectively. These results highlight the importance of understanding e-cigarette use not as a stand-alone product, but rather as part of a complex tobacco product landscape.

While e-cigarette use increased overall between 2014 and 2018 for HS students (13.4–20.8%), it did not for MS students (3.9–4.9%). These results belie differential patterns by smoking status and e-cigarette use frequency. Among MS students, there were significant increases over this period for never and current smokers but not for former smokers (although the 2019 data suggest a much higher prevalence for MS former smokers). Among HS students, increases in prevalence of use were seen for all frequency categories for never and former smokers. Among current smokers, however, the increase has been almost entirely among frequent users. This result underscores differential e-cigarette use behavior for dual users of cigarettes and e-cigarettes compared to exclusive cigarette users, and may reflect differential nicotine dependence. These results also highlight that interventions intended to reduce e-cigarette use will probably need to distinctly target smokers and non-smokers.

Interventions need to consider which groups are most at risk, which requires consideration of both numbers of people and magnitudes of potential harm. Across all survey years, the vast majority of HS never smokers did not use e-cigarettes at all or used e-cigarette infrequently. Frequent use of e-cigarettes among never smoker HS students continues to be rare. Nevertheless, the proportion of HS never smokers who used e-cigarettes frequently increased more than threefold from 2017 to 2018, representing an increase from 50 000 students in 2017 and to 170 000 by 2018. The number of corresponding HS current smokers using e-cigarettes frequently more than doubled from 160 000 to 370 000. Thus, despite the lower prevalence of frequent use among never smokers, the rate of change in their absolute numbers surpassed that of current smokers by 2018. Researchers attribute 2017–18 increases to rising JUUL use in particular [2,3,26]. With the 2019 NYTS redesign and inclusion of JUUL in its questionnaire, an estimated 420 000 HS never smokers were using e-cigarettes frequently, surpassing the 390 000 of HS frequent users who also smoked cigarettes. Hence, e-cigarette initiation among never smokers remains an acute concern. The extent to which these never smoker youth are nicotine-dependent is unclear, although other research on cigarettes, cigars and smokeless tobacco have found frequency of use to be associated with symptoms of tobacco dependence [27].

Health authorities also need to consider the different potential harms of e-cigarettes to never and current smokers. E-cigarette use could reduce the harms of combustible tobacco for current smokers if it were associated with reduced cigarette smoking, but could also interfere with quit attempts by maintaining nicotine addiction. E-cigarettes may cause nicotine addiction in never smokers and could potentially catalyze initiation to combustible products, yet rising e-cigarette use is occurring simultaneously with decreases in smoking prevalence [28,29]. More research is needed to understand the real-world impact of e-cigarettes on youth never versus current smokers. US public health authorities have expressed concern about the impact of nicotine on the developing brains of young people, regardless of its source [30]. E-cigarette users who are current smokers are already exposed to high levels of nicotine through their smoking, while the majority of never-smoking youth use e-cigarettes infrequently and probably have limited or at least lower levels of nicotine exposure compared to the smaller fraction of never users who use e-cigarettes frequently. Frequency of e-cigarette use does not correspond perfectly with nicotine exposure, given the diversity of e-cigarette devices that exist and patterns of use that vary from person to person. However, frequency measures for e-cigarette use among adolescents have been shown to correlate with cotinine, a biomarker for nicotine exposure [31]. The 490 000 HS never smokers using e-cigarettes on more than 20 days of the month in 2019 may be experiencing nicotine addiction and should be considered targets for public health interventions. These should complement ongoing efforts to help the 5.8% (860 000) and 2.3% (270 000) of HS and MS smokers to quit [32].

Nicotine exposure among never smoker youth is changing due to both the rise in ever and frequent use of e-cigarettes and new developments in the e-cigarette market-place. The average nicotine concentration in e-cigarettes sold has increased over time [33], and the e-cigarette market has shifted towards advanced, pod-based products that have been shown to deliver nicotine more efficiently to its users compared to early-generation ‘cig-alike’ products [34,35]. Pod-based e-cigarettes, such as JUUL, have become more widely used and social media content promoting such products have proliferated [36,37]. The rise of these nicotine pod-based e-cigarettes could explain the increase in e-cigarette uptake among non-smokers. JUUL Laboratories’ sales have increased rapidly, and they have the greatest market share of any e-cigarette company [38]. Many young people who use JUUL are not aware that the product contains nicotine [39], and may be at risk for nicotine dependence.

Cigarette smoking by youth has been declining, but some e-cigarette users may be using other types of combustible tobacco products, particularly cigars [40]. Our findings confirm that current and frequent e-cigarette use remains concentrated among combustible tobacco users. A separate NYTS analysis reports that although combustible tobacco use has continued to decline from 2014 to 2018, a rising share are co-using with e-cigarettes among remaining users [29]. Similarly, ever and current use of e-cigarettes among both never and former combustible tobacco users has also increased. However, the proportion of youth using any tobacco product (including e-cigarettes) in the past 30 days did not change significantly from 2014 to 2018 [29], which suggests that e-cigarettes could be displacing other forms of tobacco among young people.

This study is subject to several limitations. Changes in survey methodology made it not possible to compare the 2014–18 data to the 2019 data directly, making it difficult to assess exactly how patterns of e-cigarette use are changing. Ongoing surveillance efforts from 2020 on-wards can identify whether recent trends among each smoking group will continue, plateau or subside. In addition, the cross-sectional nature of the analysis prohibits claims about whether use e-cigarettes among never smoker youth leads to greater or lower risk of subsequent smoking initiation. While longitudinal research may support the former, other trend studies indicate that, on aggregate, e-cigarette use does not appear to be increasing smoking rates [28,41–44]. If adolescent smoking rates continue to decline as they have been, it is possible that rising e-cigarette use could be diverting non-smoking youth away from cigarettes.

Our estimates indicate that a rising share of young never and former smokers are using e-cigarettes and that the overwhelming majority of current smoker youth are dual-using with e-cigarettes. If young current or former smokers (never smokers) are gradually switching to e-cigarettes rather than continuing (starting) to smoke, this would reduce the net total population harm caused by tobacco. However, realization of harm reduction depends upon whether never smokers using e-cigarettes are individuals who would have otherwise started smoking cigarettes if e-cigarettes did not exist. Future research following the tobacco use trajectories of never smokers who use e-cigarettes frequently may help to determine whether this growing minority of users is decelerating declines in youth smoking.

The national e-cigarette policy context can greatly influence patterns of adolescent use. For example, youth e-cigarette use has apparently stabilized in the United Kingdom, where e-cigarette companies face greater marketing restrictions [45]. Since 2014, the European Union has placed rules on the manufacture of e-cigarette products, including limits on the levels of nicotine in e-liquids to no more than 20 mg/ml [46]. In contrast, e-cigarettes did not come under the US FDA regulatory jurisdiction until 2016 and were not required to undergo FDA product review until September 2020, despite the products having already existed on the US market for several years [47]. The US experience with respect to adolescent e-cigarette use differs from that of other countries, due probably to these and other differences related to policy and social contexts.

Ongoing efforts from governments and health organizations could address rising e-cigarette use among never smoker youth in the United States. Recent media campaigns highlighting the potential risks associated with e-cigarettes could reduce youth e-cigarette use or slow their increased uptake [48]. Since 2018 schools have started to address e-cigarette use among students [49], and enforcement efforts have targeted retailers in violation of minimum age laws [50]. While the US FDA has removed cartridge-based e-cigarette flavors (except for tobacco and menthol) from the market [15], it is possible that youth could still switch to non-cartridge-based e-cigarettes that remain available in sweet or sugary flavors. These initiatives, growing concerns about e-cigarette-related lung injuries attributed to THC use [13,51], and the disrupting effects of COVID-19 are probably changing this trajectory, as newer data show that current e-cigarette use among adolescents decreased from 2019 to 2020 [52]. Whether these and other public health efforts translate into fewer never smokers using e-cigarettes beyond 2020 remains to be seen.

## Supplementary Material

Tam 2021 Supplement Table S1 Current e-cigarette use among high school and middle school students by combustible tobacco use status, National Youth Tobacco Survey 2014–2019.
Table S2 Past 30-day e-cigarette use frequency among high school students by combustible tobacco use status, National Youth Tobacco Survey 2014–2019.
Table S3 Past 30-day e-cigarette use frequency among middle school students by combustible tobacco use status, National Youth Tobacco Survey 2014–2019.

## Figures and Tables

**Table 1:** Characteristics of high school and middle school students, National Youth Tobacco Surveys 2014–19.

Survey year	High school						Middle school					
	2014	2015	2016	2017	2018	2019^^^	2014	2015	2016	2017	2018	2019^^^
Unweighted *n*	11 399	9433	10 897	10 186	10991	10 097	10 419	8170	9658	7562	9055	8837
Weighted *n*	15 194 249	15 156 493	15 123 092	15 097441	14 988 762	15 044 983	11902 292	11968 188	11975 780	11887910	11 845 323	11860123
Sex (%)												
Female	5536(49.7)	4516 (51.2)	5383 (49.9)	5025 (51.5)	5398 (49.8)	4766 (52.5)	5093 (50.8)	4085 (51.2)	4680(51.6)	3770(51)	4501 (51.4)	4310 (51.3)
Male	5834(50.3)	4889 (48.8)	5479(50.1)	5105 (48.5)	5539 (50.2)	5291 (47.5)	5289 (49.2)	4044 (48.8)	4937 (48.4)	3752 (49)	4508 (48.6)	4471 (48.7)
Missing	29	28	35	56	54	40	37	41	41	40	46	56
Age, years (%)												
9	17 (0.1)	7(0.1)	18 (0.2)	10 (0.1)	23 (0.2)	12 (0.1)	21 (0.1)	7 (0)	20 (0.2)	15 (0.1)	28 (0.2)	22 (0.2)
10	1 (0)	3 (0)	0 (0)	6 (0)	1 (0)	4 (0)	10 (0.1)	1 (0)	7 (0.1)	6 (0.1)	5 (0)	15 (0.2)
11	0 (0)	1 (0)	3 (0)	2 (0)	2 (0)	4 (0)	1324(12.2)	926(11.1)	1291 (12.3)	1016(13.7)	1005 (11.6)	1098 (12)
12	1 (0)	3 (0)	2 (0)	3 (0)	4 (0)	7 (0.1)	3076 (30)	2442 (30.7)	2961 (30.8)	2408 (31.6)	2869 (31.3)	2768 (30.8)
13	8 (0.1)	4 (0)	5 (0)	6 (0)	10 (0.1)	7 (0)	3600 (34.7)	2826 (33.6)	3187 (34.4)	2488 (33.4)	2998 (32.8)	3061 (34.5)
14	1097(10.2)	951 (10.2)	1126 (11.3)	1006 (10.8)	1012 (9.6)	1068 (10.3)	2117(20.4)	1781 (22.3)	1947 (19.8)	1470(19.5)	1913 (21.7)	1734(21)
15	2699 (24.9)	2376 (25.4)	2 577(24.8)	2573 (26.7)	2682 (25.4)	2471 (24.5)	226 (2.1)	156 (2.1)	201 (2.2)	122 (1.4)	190 (2.1)	115 (1.2)
16	2876(25)	2421 (24.5)	2851 (25.9)	2569(25.1)	2769 (25.4)	2510(25.5)	13 (0.2)	13 (0.1)	12 (0.2)	13 (0.1)	13 (0.1)	3 (0)
17	2837(24.1)	2298 (24.5)	2675 (23.5)	2514(23.4)	2710(23.4)	2 523 (24.4)	5 (0)	1 (0)	3 (0)	5 (0.1)	6 (0)	1 (0)
18	1675 (14.3)	1245 (14)	1484 (13.2)	1359 (12.9)	1624(14.5)	1371 (14)	3 (0.1)	3 (0)	1 (0)	0 (0)	2 (0)	1 (0)
19+	170 (1.3)	118(1.2)	147(1.2)	129 (0.9)	146 (1.4)	112 (1.1)	4 (0)	6 (0)	9 (0.1)	6 (0)	9 (0.1)	6 (0)
Missing	18	6	9	9	8	8	20	8	19	13	17	13
Grade (%)												
6th							3357(31.6)	2552 (33.2)	3235 (33.1)	2 524 (33.1)	2903 (33.2)	2944 (33.2)
7th							3541 (34.2)	2845 (33.1)	3249 (33.5)	2565 (33.5)	3140(33.4)	3024 (33.3)
8th							3521 (34.1)	2773 (33.7)	3174 (33.4)	2473 (33.5)	3012 (33.4)	2869 (33.5)
9th	2885 (27.2)	2512 (27.3)	2741 (27.4)	2583 (27.2)	2935 (27.3)	2790 (27.4)						
10th	2933 (25.5)	2 509 (25.7)	2809(25.7)	2637(25.8)	2664(25.7)	2499(25.7)						
11th	2817(24)	2282 (23.9)	2674(23.9)	2575 (23.9)	2824(23.9)	2 502 (23.9)						
12th	2764(23.3)	2130 (23.1)	2673 (23)	2391 (23.1)	2568 (23.2)	2306 (23.1)						
Race/Hispanic origin (%)												
NH-white	5407(59)	4695 (57.9)	4805 (56.8)	4755 (58.2)	5647(57.6)	5129 (57.3)	4473 (57.6)	3775 (55.6)	4162 (55.3)	3498(55.6)	4119 (54.4)	4200(55)
NH-black	1899 (16)	1357(14.1)	1828 (13.2)	1816 (12.7)	1365 (12.3)	12 56 (13.2)	1520 (14.4)	1180 (14.4)	1395 (12.9)	1290 (13.5)	1176(13.7)	1166 (13.4)
Hispanic	2975 (20.6)	2654 (23.1)	3100(24.6)	2653 (23.5)	3016 (23.6)	2897 (24)	3069 (23.5)	2157(24.4)	2665 (26.5)	1931 (25.2)	2712 (26.3)	2639 (26.1)
NH-Asian	590 (3.6)	272 (3.2)	603 (4.3)	510 (4.6)	458 (5.3)	560 (4.3)	380(3.5)	400 (3.9)	481 (3.8)	242 (4.2)	293 (4.1)	334 (4)
NH-AI/AN	100(0.5)	97(0.6)	130 (0.6)	82 (0.6)	116 (0.6)	87(0.6)	237 (0.8)	136 (1.1)	290 (0.8)	151 (0.9)	187 (0.9)	136 (0.8)
NH-NHOPI	51 (0.3)	60 (1)	83 (0.5)	51 (0.4)	56 (0.6)	53 (0.6)	34 (0.3)	56 (0.5)	75 (0.7)	45 (0.6)	44 (0.6)	50 (0.8)
Missing	377	298	348	319	333	115	706	466	590	405	524	312
Smoking status (%)												
Never smoker	7604(70.1)	6443 (70.3)	7865 (73.2)	7641 (77.6)	8056 (76.9)	7919 (77.5)	9028 (89.5)	7201 (90.1)	8461 (90.4)	6749 (92.4)	8146 (92.9)	8081 (91.7)
Current smok	er 1104(9.3)	873 (9.3)	772 (8.1)	761 (7.6)	912 (8.2)	549 (5.8)	273 (2.5)	194 (2.3)	193 (2.2)	194 (2.1)	169 (1.8)	190(2.3)
Former smoke	T 2379 (20.6)	1860 (20.3)	1949 (18.7)	1506 (14.8)	1702 (14.9)	1609 (16.7)	818 (8)	589 (7.6)	714(7.4)	437(5.4)	508 (5.4)	544 (6)
Missing	312	257	311	278	321	20	300	186	290	182	232	22
Past 30-day e-cigarette use (%)												
No	−9724 (86.6)	7843 (84)	9603 (88.7)	8949 (88.3)	8539 (79.2)	7333 (72.5)	9752 (96.1)	7575 (94.7)	9078 (95.7)	7187(96.7)	8436(95.1)	7894(89.5)
Yes	1505 (13.4)	1469 (16)	1109(11.3)	1066(11.7)	2227(20.8)	2709 (27.5)	487 (3.9)	471 (5.3)	392 (4.3)	272 (3.3)	454 (4.9)	902 (10.5)
Missing	170	121	185	171	225	55	180	124	188	103	165	41
Combustible tobacco use status (%)												
Never	6439(58) 5456(58.5)	6887(63.5)	6804 (67.8)	7177(66.9)	6930 (67.9)	8748 (84.8)	7020 (86.4)	8275 (86.3)	6636 (89.6)	7992 (89.2)	7667 (87)
Current	2161 (18.1)	1682 (17.2)	1503 (13.8)	1369 (12.9)	1588 (13.9)	1167(12)	576 (5.1)	367(4)	413 (4.3)	318 (3.3)	318 (3.3)	403 (4.8)
Former	2776 (24)	2274 (24.3)	2480(22.7)	1990(19.3)	2197 (19.2)	1993 (20.1)	1076 (10.1)	765 (9.6)	959 (9.4)	603 (7.1)	726 (7.6)	761 (8.2)
Missing	23	21	27	23	29	7	19	18	11	5	19	6

Numbers in cells represent number of respondents in each survey sample. Percentages in parentheses (%) represent weighted estimates which may not sum to 100% due to rounding. Missing observations are excluded from weighted percentage estimates reported in parentheses. NH = non-Hispanic; AI/AN = American Indian/Alaska Native; NHOPI = Native Hawaiian or Other PacificIslander.

^ Data from 2019 are not directly comparable with estimates in previous years due to changes in the National Youth Tobacco Survey (NYTS) format from paper to electronic. Combustible tobacco includes cigarettes (including roll-your-own), cigars (cigars, cigarillos, little cigars), pipe tobacco, hookah or bidis. Never combustible users report never using combustible tobacco products, not even ‘one or two puffs’. Former combustible users report ever using combustible tobacco products, even ‘one or two puffs’, but no use in the past 30 days. Current combustible users report using any combustible tobacco product in the past 30 days.

**Table 2:** Current e-cigarette use among high school and middle school students by smoking status, National Youth Tobacco Survey 2014–19.

Year	All	n	Wtd n	Never smokers	n	Wtd n	Former smokers	n	Wtd n	Current smokers	n	Wtd n
High school												
2014	13.4 (11.0–15.9)	1505	2 010 000	4.7 (3.5–5.9)	359	480 000	23.0(18.4–27.6)	510	690 000	56.6 (49.4–63.8)	576	760 000
2015	16.0(14.0–17.9)	1469	2 390 000	6.9* (5.8–8.0)	441	700 000	27.9 (23.8–31.9)	479	820 000	55.9(48.8–62.9)	480	740 000
2016	11.3* (9.8–12.8)	1109	1 680 000	4.6* (3.6–5.6)	339	480 000	19.5* (16.9–22.0)	336	520 000	52.6 (47.1–58.0)	381	600 000
2017	11.6 (9.6–13.8)	1066	1 730 000	5.3 (4.0–6.5)	354	590000	21.8 (17.4–26.2)	280	470 000	52.4 (45.5–59.3)	370	580 000
2018	20.8* (18.8–22.8)	2227	3 050 000	11.7* (10.1–13.2)	892	1280 000	38.9* (34.0–43.9)	634	830 000	71.0* (66.1–75.9)	609	810 000
2019^a^	27.5^a^ (25.3–29.7)	2709	4110 000	17.5^a^ (16.0–19.0)	1367	2 020 000	53.6^a^ (45.2–61.9)	874	1 330 000	85.8^a^ (81.6–89.9)	466	740 000
Middle school												
2014	3.9 (2.9–4.8)	487	450 000	1.4 (1.0–1.9)	154	140 000	14.6 (10.2–18.9)	133	130 000	45.8 (30.6–60.9)	140	120 000
2015	5.3* (4.5–6.1)	471	620 000	2.3* (1.8–2.8)	200	240 000	19.8 (15.5–24.1)	120	170000	60.1 (49.4–70.8)	116	150 000
2016	4.3* (3.6–4.9)	392	500 000	1.7* (1.3–2.0)	135	170 000	16.2 (13.1–19.3)	113	130 000	57.8 (46.9–68.8)	108	140 000
2017	3.3* (2.8–3.9)	272	390 000	1.3 (1.0–1.7)	94	140 000	12.0 (8.7–15.2)	49	70 000	58.6 (48.0–69.1)	102	130 000
2018	4.9* (4.1–5.7)	454	570 000	2.8* (2.2–3.4)	234	290 000	14.6 (10.5–18.7)	85	80 000	72.8* (65.4–80.2)	102	140 000
2019^a^ (9.3–11.8)	902	1 240 000	6.8^a^ (5.9–7.7)	540	730 000	40.8^a^ (34.7–47.0)	217	280 000	78.0^a^ (71.9–84.2)	140	210 000

Numbers in parentheses represent 95% confidence intervals. Wtd = weighted. Estimated total number of users was rounded down to the nearest 10 000 people. Current use of e-cigarettes was defined as any use of e-cigarettes during the past 30 days. Smoking status is assessed with the question: ‘Have you ever tried cigarette smoking, even one or two puffs?’. ‘Never smokers’ are those who responded ‘no’ and ‘former smokers’ are those who responded ‘yes’, but report no smoking in the past 30 days. Youth who have smoked at all within the past 30 days are considered to be ‘current smokers’.

* *P*-value < 0.05 for the difference in prevalence compared to the previous year in a general linear model simultaneously testing the effect of smoking status, year and the interaction between smoking status and year.

a Data from 2019 are not directly comparable with estimates in previous years due to changes in the National Youth Tobacco Survey (NYTS) format from paper to electronic.

**Table 3:** Past 30-day e-cigarette use frequency among high school students by smoking status, National Youth Tobacco Survey 2014–19.

Year	None	n	Wtd n	Infrequent^a^	n	Wtd n	Moderate	n	Wtd n	Frequent	n	Wtd n
Never smokers												
2014	95.3 (94.2 96.5)	7173	9 810 000	3.9 (3.0–4.8)	290	400 000	0.6 (0.3–0.8)	52	60 000	-^b^		-
2015	93.1* (92.0–94.2)	5954	9 570 000	5.1* (4.2–6.0)	329	520 000	1.4* (0.9–1.8)	86	140 000	0.5* (0.3–0.7)	26	40 000
2016	95.4* (94.4–96.4)	7449	10190 000	3.5* (2.7–4.3)	260	370 000	0.8* (0.5–1.1)	56	80 000	0.3 (0.1–0.4)	23	20 000
2017	94.7(93.5–96.0)	7201	10 720 000	4.1 (3.2–5.1)	277	460 000	0.6 (0.3–1.0)	46	70 000	0.5 (0.3–0.8)	31	50 000
2018	88.3* (86.8–89.9)	7064	9 760 000	7.5* (6.5–8.5)	571	820 000	2.6* (2.0–3.2)	191	280000	1.6* (1.1–2.1)	130	170000
2019^c^	82.5^c^ (81.0–84.0)	6518	9 560 000	10.5^c^ (9.6–11.4)	819	1210 000	3.3^c^ (2.8–3.8)	259	380 000	3.7^c^ (3.0–4.3)	290	420 000
Former smokers												
2014	77.0 (72.4–81.6)	1848	2 330 000	14.6 (11.7–17.4)	329	440 000	5.6 (3.9–7.3)	124	160 000	2.9 (1.6–4.1)	57	80 000
2015	72.1 (68.0–76.2)	1364	2 140 000	17.2 (14.4–19.9)	311	510 000	7.5 (5.3–9.7)	116	220 000	3.2 (1.9–4.4)	52	90 000
2016	80.5* (78.0–83.1)	1584	2 180 000	12.5* (10.6–14.4)	219	330 000	3.5* (2.5–4.6)	66	90 000	3.4 (2.2–4.7)	51	90 000
2017	78.2 (73.8–82.6)	1207	1680 000	13.2 (10.4–15.9)	173	280000	4.4 (3.1–5.7)	60	90 000	4.2 (2.3–6.2)	47	90 000
2018	61.1* (56.1–66.0)	1045	1 300 000	17.5* (15.2–19.7)	300	370 000	9.6* (7.5–11.6)	148	200000	11.9* (9.1–14.6)	186	250 000
2019^c^	46.4^c^ (38.1–54.8)	729	1 160 000	19.3^c^ (16.0–22.7)	323	480 000	11.I^c^ (8.8–13.3)	179	270000	23.2^c^ (18.1–28.2)	372	570 000
Current smokers												
2014	43.4(36.2–50.7)	504	580 000	26.6 (22.5–30.8)	280	350 000	16.2 (12.6–19.7)	160	210 000	13.8 (9.3–18.3)	136	180 000
2015	44.1 (37.0–51.2)	362	580 000	27.7(24.1–31.4)	232	360 000	12.6 (10.1–15.0)	116	160 000	15.5 (10.9–20.2)	132	200 000
2016	47.4(42.0–52.9)	361	540 000	27.0(21.9–32.1)	186	300 000	11.7(9.0–14.4)	86	130 000	13.8 (10.1–17.5)	109	150 000
2017	47.6(40.8–54.5)	376	520 000	23.1 (19.8–26.5)	164	250 000	14.0(9.8–18.2)	91	150 000	15.2 (9.9–20.5)	115	160 000
2018	29.0* (24.1–33.9)	273	330 000	22.1 (18.2–26.1)	184	250 000	16.1 (12.6–19.6)	140	180 000	32.7* (26.8–38.6)	285	370000
2019^c^	14.2^c^ (10.1–18.4)	81	120 000	24.2^c^ (20.5–28.0)	124	200000	15.4^c^ (11.8–19.1)	88	130 000	46.1^c^ (39.1–53.2)	254	390 000

Numbers in parentheses represent 95% confidence intervals. Wtd = weighted. Estimated total number of users was rounded down to the nearest 10 000 people. Past 30-day use of e-cigarettes was determined by asking: ‘During the past 30 days, on how many days did you use e-cigarettes?’. Smoking status is assessed with the question: ‘Have you ever tried cigarette smoking, even one or two puffs?’. ‘Never smokers’ are those who responded ‘no’ and ‘former smokers’ are those who responded ‘yes’, but report no smoking in the past 30 days. Youth who have smoked at all within the past 30 days are considered to be ‘current smokers’.

* *P*-value < 0.05 for the difference in prevalence compared to the previous year in a general linear model simultaneously testing the effect of smoking status, year and the interaction between smoking status and year.

a Infrequent = used e-cigarettes 1–5daysinthepast30days;moderate=6–19 days; frequent = 20–30 days.

b Dashes indicate estimates that are unreliable because the relative standard error was *>* 30% or the unweighted denominator was < 50.

c Data from 2019 are not directly comparable with estimates in previous years due to changes in the National Youth Tobacco Survey (NYTS) format from paper to electronic.

**Table 4:** Past 30-day e-cigarette use frequency among middle school students by smoking status, National Youth Tobacco Survey 2014–2019.

Year	None	n	Wtd n	Infrequent^a^	n	Wtd n	Moderate or frequent	n	Wtd n
Never smokers									
2014	98.5 (98.1–99.0)	8762	10110 000	1.3 (0.9–1.7)	131	130 000	0.2 (0.1–0.3)	23	10 000
2015	97.7* (97.2–98.2)	6913	10150 000	1.9* (1.5–2.3)	160	190 000	0.4 (0.2–0.6)	40	40 000
2016	98.3* (98.0–98.7)	8194	10190 000	1.4* (1.0–1.7)	108	140 000	0.3 (0.2–0.4)	27	30 000
2017	98.7 (98.3–99.0)	6589	10 540 000	1.2 (0.8–1.5)	79	120 000	-^b^	-	-
2018	97.2* (96.6–97.8)	7801	10 280 000	2.1*(1.7–2.6)	184	220 000	0.6* (0.3–0.9)	50	60 000
2019^c^	93.2^c^ (92.2–94.1)	7513	10 080 000	5.1^c^ (4.3–5.8)	399	550 000	1.8^c^ (1.4–2.1)	143	180 000
Former smokers									
2014	85.4(81.0–89.8)	668	760 000	10.9 (7.4–14.5)	96	90 000	3.6 (1.8–5.4)	37	30 000
2015	80.2 (76.0–84.5)	460	700 000	15.6 (11.5–19.6)	89	130 000	4.2 (2.5–5.9)	31	30 000
2016	83.8 (80.7–86.9)	585	710 000	10.8 (8.0–13.5)	77	90 000	5.4 (3.4–7.5)	36	40 000
2017	88.0(84.8–91.3)	382	550000	9.1 (6.0–12.3)	40	50 000	-	-	-
2018	85.4(81.3–89.5)	415	510 000	8.9(5.6–12.3)	53	50 000	5.7* (3.2–8.1)	32	30 000
2019^c^	59.2^c^ (53.0–65.3)	326	410 000	22.9^c^ (18.4–27.4)	126	160 000	17.9^c^ (12.8–23.1)	91	120 000
Current smokers									
2014	54.2 (39.1–69.4)	117	150 000	23.2 (15.3–31.0)	78	60 000	22.6 (12.6–32.5)	62	60 000
2015	39.9 (29.3–50.5)	74	100 000	29.0 (19.7–38.3)	53	70 000	31.1 (22.1–40.1)	63	70 000
2016	42.2 (31.3–53.1)	79	100 000	28.2 (19.1–37.4)	51	70 000	29.6 (22.1–37.1)	57	70 000
2017	41.4 (30.9–52.0)	84	90 000	28.9 (17.4–40.4)	52	60 000	29.7(20.4–39.0)	50	70 000
2018	2 7.2* (19.8–34.6)	58	50 000	29.4 (21.7–37.1)	44	50 000	43.4* (33.5–53.3)	58	80 000
2019^c^	22.0^c^ (15.8–28.1)	47	50 000	36.4^c^ (28.7–44.1)	63	90 000	41.7^c^ (32.4–50.9)	77	110 000

Numbers in parentheses represent 95% confidence intervals. Wtd = weighted. Estimated total number of users was rounded down to the nearest 10 000 people. Past 30-day use of e-cigarettes was determined by asking: ‘During the past 30 days, on how many days did you use e-cigarettes?’. Smoking status is assessed with the question: ‘Have you ever tried cigarette smoking, even one or two puffs?’. ‘Never smokers’ are those who responded ‘no’ and ‘former smokers’ are those who responded ‘yes’, but report no smoking in the past 30 days. Youth who have smoked at all within the past 30 days are considered to be ‘current smokers’.

* *P*-value < 0.05 for the difference in prevalence compared to the previous year in a general linear model simultaneously testing the effect of smoking status, year, and the interaction between smoking status and year.

a Infrequent = used e-cigarettes 1–5 days in the past 30 days; moderate or frequent = 6–30 days.

b Dashes indicate estimates that are unreliable because the relative standard error was *>* 30% or the unweighted denominator was < 50.

c Data from 2019 are not directly comparable with estimates in previous years due to changes in the National Youth Tobacco Survey (NYTS) format from paper to electronic.
