# Supplementary material for: Comparison of e-cigarette use prevalence and frequency by smoking status among youth in the United States, 2014–19
Source: Addiction. Author manuscript; Available in PMC 2021 Sep 1. (PMC8328922; doi:10.1111/add.15439)
Supplement: Tam 2021 Supplement — Table S1 Current e-cigarette use among high school and middle school students by combustible tobacco use status, National Youth Tobacco Survey 2014–2019. Table S2 Past 30-day e-cigarette use frequency among high school students by combustible tobacco use status, National Youth Tobacco Survey 2014–2019. Table S3 Past 30-day e-cigarette use frequency among middle school students by combustible tobacco use status, National Youth Tobacco Survey 2014–2019. [file NIHMS1698910-supplement-Tam_2021_Supplement.docx]

**Table S1. Current e-cigarette use among high school and middle school students by combustible tobacco use status, National Youth Tobacco Survey 2014-2019**

| **Year** | **All** | **n** | **wtd n** | **Never**  **combustible user** | **n** | **wtd n** | **Former**  **combustible user** | **n** | **wtd n** | **Current**  **combustible**  **user** | **n** | **wtd n** |
| --- | --- | --- | --- | --- | --- | --- | --- | --- | --- | --- | --- | --- |
| **High school** | | | | | | | | | | | | |
| **2014** | 13.4 (11.0, 15.9) | 1505 | 2,010,000 | 2.6 (1.6, 3.6) | 172 | 220,000 | 14.7 (11.2, 18.2) | 398 | 530,000 | 46.9 (40.7, 53.1) | 935 | 1,250,000 |
| **2015** | 16.0 (14.0, 17.9) | 1469 | 2,390,000 | 3.8 (2.9, 4.7) | 200 | 330,000 | 17.9 (14.8, 21.0) | 376 | 650,000 | 55.0* (50.0, 60.1) | 891 | 1,400,000 |
| **2016** | 11.3* (9.8, 12.8) | 1109 | 1,680,000 | 2.9 (2.2, 3.6) | 193 | 270,000 | 14.6 (12.0, 17.2) | 316 | 490,000 | 45.1* (40.8, 49.6) | 598 | 900,000 |
| **2017** | 11.6 (9.6, 13.8) | 1066 | 1,730,000 | 3.3 (2.3, 4.3) | 207 | 330,000 | 18.7 (15.2, 22.2) | 302 | 530,000 | 45.5 (39.6, 51.3) | 556 | 860,000 |
| **2018** | 20.8* (18.8, 22.8) | 2227 | 3,050,000 | 9.0* (7.5, 10.5) | 622 | 890,000 | 32.9* (27.8, 37.9) | 683 | 930,000 | 61.9* (57.4, 66.5) | 921 | 1,230,000 |
| **2019^^^** | 27.5 (25.3, 29.7) | 2709 | 4,110,000 | 13.8 (12.4, 15.2) | 933 | 1,400,000 | 45.6 (38.6, 52.5) | 914 | 1,360,000 | 74.8 (69.9, 79.6) | 862 | 1,340,000 |
| **Middle school** | | | | | | | | | | | | |
| **2014** | 3.9 (2.9, 4.8) | 487 | 450,000 | 1.1 (0.7, 1.5) | 109 | 100,000 | 10.5 (6.7, 14.3) | 134 | 120,000 | 39.7 (30.3, 49.0) | 243 | 220,000 |
| **2015** | 5.3 (4.5, 6.1) | 471 | 620,000 | 1.6 (1.2, 1.9) | 121 | 150,000 | 14.9 (11.6, 18.2) | 121 | 160,000 | 63.9* (56.1, 71.7) | 227 | 290,000 |
| **2016** | 4.3 (3.6, 4.9) | 392 | 500,000 | 1.2 (0.8, 1.5) | 94 | 110,000 | 13.1 (9.8, 16.4) | 107 | 140,000 | 48.3* (41.6, 55.0) | 191 | 230,000 |
| **2017** | 3.3 (2.8, 3.9) | 272 | 390,000 | 1.1 (0.8, 1.5) | 73 | 110,000 | 9.4 (5.7, 13.2) | 57 | 70,000 | 49.9 (40.4, 59.4) | 140 | 180,000 |
| **2018** | 4.9* (4.1, 5.7) | 454 | 570,000 | 2.3* (1.8, 2.8) | 185 | 230,000 | 16.0* (12.4, 19.6) | 121 | 130,000 | 53.8 (45.9, 61.7) | 148 | 190,000 |
| **2019^^^** | 10.5 (9.3, 11.8) | 902 | 1,240,000 | 5.2 (4.4, 6.1) | 402 | 540,000 | 33.3 (27.9, 38.7) | 244 | 320,000 | 69.2 (63.3, 75.1) | 256 | 380,000 |

Numbers in parentheses represent 95% confidence intervals.

Wtd = weighted. Estimated total number of users was rounded down to the nearest 10,000 persons.

*p-value <0.05 for the difference in prevalence compared to the previous year in a general linear model simultaneously testing the effect of smoking status, year, and the interaction between smoking status and year.

Current use of e-cigarettes was defined as any use of e-cigarettes during the past 30 days.

Combustible tobacco includes cigarettes (including roll-your-own), cigars (cigars, cigarillos, little cigars), pipe tobacco, hookah, or bidis.

Never combustible users report never using any combustible tobacco products, not even “one or two puffs”.

Former combustible users report ever using any combustible tobacco products, even “one or two puffs”, but no use in the past 30 days.

Current combustible users report using any combustible tobacco product in the past 30 days.

^^^Data from 2019 are not directly comparable with estimates in previous years due to changes in the NYTS format from paper to electronic.

**Table S2. Past 30-day e-cigarette use frequency among high school students by combustible tobacco use status, National Youth Tobacco Survey 2014-2019**

| **Year** | **None** | **n** | **wtd n** | **Infrequent**^a^ | **n** | **wtd n** | **Moderate** | **n** | **wtd n** | **Frequent** | **n** | **wtd n** |
| --- | --- | --- | --- | --- | --- | --- | --- | --- | --- | --- | --- | --- |
| **Never combustible user** | | | | | | | | | | | | |
| **2014** | 97.4 (96.4, 98.4) | 6196 | 8,470,000 | 2.3 (1.4, 3.1) | 147 | 190,000 | 0.3 (0.1, 0.4) | 20 | 20,000 | —^§§^ |  | — |
| **2015** | 96.2* (95.3, 97.1) | 5209 | 8,430,000 | 3.0 (2.2, 3.7) | 157 | 250,000 | 0.6* (0.4, 0.9) | 32 | 50,000 | — |  | — |
| **2016** | 97.1* (96.4, 97.8) | 6619 | 9,200,000 | 2.3 (1.7, 3.0) | 157 | 220,000 | 0.4 (0.2, 0.7) | 27 | 40,000 | — |  | — |
| **2017** | 96.7 (95.7, 97.7) | 6517 | 9,770,000 | 2.7 (1.9, 3.5) | 170 | 270,000 | 0.3 (0.1, 0.5) | 22 | 30,000 | — |  | — |
| **2018** | 91.0* (89.5, 92.5) | 6452 | 8,970,000 | 6.2* (5.2, 7.2) | 428 | 610,000 | 1.8* (1.3, 2.3) | 120 | 170,000 | 1.1* (0.7, 1.4) | 74 | 100,000 |
| **2019^^^** | 86.2 (84.8, 87.5) | 5971 | 8,760,000 | 9.0 (8.1, 10.0) | 619 | 910,000 | 2.5 (2.1, 3.0) | 167 | 250,000 | 2.2 (1.8, 2.8) | 148 | 220,000 |
| **Former combustible user** | | | | | | | | | | | | |
| **2014** | 85.3 (81.8, 88.8) | 2350 | 3,070,000 | 10.4 (8.1, 12.7) | 285 | 370,000 | 2.9 (1.9, 3.9) | 81 | 100,000 | 1.4 (0.6, 2.2) | 32 | 50,000 |
| **2015** | 82.1* (79.0, 85.2) | 1876 | 2,990,000 | 12.2 (10.4, 14.1) | 271 | 440,000 | 3.6 (2.1, 5.2) | 71 | 130,000 | 2.0 (1.1, 2.9) | 34 | 70,000 |
| **2016** | 85.4* (82.8, 88.0) | 2129 | 2,880,000 | 9.8 (7.9, 11.7) | 221 | 330,000 | 3.1 (2.2, 4.0) | 60 | 100,000 | 1.6 (0.9, 2.4) | 35 | 50,000 |
| **2017** | 81.3* (77.8, 84.8) | 1657 | 2,320,000 | 12.5 (10.4, 14.6) | 208 | 350,000 | 3.4 (2.2, 4.7) | 54 | 90,000 | 2.8 (1.5, 4.0) | 40 | 70,000 |
| **2018** | 67.1* (62.1, 72.2) | 1484 | 1,900,000 | 16.0* (13.6, 18.4) | 347 | 450,000 | 8.5* (6.2, 10.7) | 161 | 230,000 | 8.4* (6.0, 10.8) | 175 | 230,000 |
| **2019^^^** | 54.4 (47.5, 61.4) | 1060 | 1,630,000 | 19.0 (15.8, 22.2) | 385 | 570,000 | 9.3 (7.4, 11.2) | 186 | 270,000 | 17.2 (13.4, 21.1) | 343 | 510,000 |
| **Current combustible user** | | | | | | | | | | | | |
| **2014** | 53.1 (46.9, 59.3) | 1174 | 1,420,000 | 25.0 (21.8, 28.1) | 500 | 670,000 | 12.4 (9.7, 15.2) | 246 | 330,000 | 9.5 (6.7, 12.3) | 189 | 250,000 |
| **2015** | 45.0* (39.9, 50.0) | 749 | 1,140,000 | 29.7* (26.6, 32.8) | 484 | 750,000 | 14.3 (11.9, 16.8) | 230 | 360,000 | 11.0 (8.1, 13.8) | 177 | 270,000 |
| **2016** | 54.9* (50.4, 59.2) | 852 | 1,100,000 | 24.7* (20.8, 28.6) | 315 | 490,000 | 9.4* (7.6, 11.1) | 134 | 180,000 | 11.1 (8.7, 13.5) | 149 | 220,000 |
| **2017** | 54.5 (48.7, 60.4) | 774 | 1,030,000 | 21.7 (19.0, 24.5) | 261 | 410,000 | 11.0 (8.6, 13.4) | 137 | 200,000 | 12.7 (8.6, 16.8) | 158 | 240,000 |
| **2018** | 38.1* (33.5, 42.6) | 602 | 750,000 | 21.1 (18.5, 23.7) | 305 | 410,000 | 15.7* (12.8, 18.6) | 230 | 310,000 | 25.2* (20.9, 29.4) | 386 | 500,000 |
| **2019^^^** | 25.2 (20.4, 30.1) | 300 | 450,000 | 23.5 (20.7, 26.3) | 262 | 420,000 | 14.6 (12.2, 16.9) | 174 | 260,000 | 36.7 (31.4, 42.1) | 426 | 650,000 |

Numbers in parentheses represent 95% confidence intervals.

Wtd = weighted. Estimated total number of users was rounded down to the nearest 10,000 persons.

*p-value <0.05 for the difference in prevalence compared to the previous year in a general linear model simultaneously testing the effect of smoking status, year, and the interaction between smoking status and year.

Past 30-day use of e-cigarettes was determined by asking, “During the past 30 days, on how many days did you use e-cigarettes?” Combustible tobacco includes cigarettes (including roll-your-own), cigars (cigars, cigarillos, little cigars), pipe tobacco, hookah, or bidis. Never combustible users report never using any combustible tobacco products, not even “one or two puffs”. Former combustible users report ever using any combustible tobacco products, even “one or two puffs”, but no use in the past 30 days. Current combustible users report using any combustible tobacco product in the past 30 days.

^§§^Dashes indicate estimates that are unreliable because the relative standard error was >30% or the unweighted denominator was < 50.

^a^Infrequent = used e-cigarettes 1-5 days in the past 30 days; Moderate = 6-19 days; Frequent = 20-30 days.

^^^Data from 2019 are not directly comparable with estimates in previous years due to changes in the NYTS format from paper to electronic.

**Table S3.** **Past 30-day e-cigarette use frequency among middle school students by combustible tobacco use status, National Youth Tobacco Survey 2014-2019**

| **Year** | **None** | **n** | **wtd n** | **Infrequent**^a^ | **n** | **wtd n** | **Moderate or Frequent** | **n** | **wtd n** |
| --- | --- | --- | --- | --- | --- | --- | --- | --- | --- |
| **Never combustible user** | | | | | | | | | |
| **2014** | 98.9 (98.5, 99.3) | 8527 | 9,850,000 | 1.0 (0.6, 1.3) | 93 | 90,000 | 0.1 (0.1, 0.2) | 16 | 10,000 |
| **2015** | 98.4 (98.1, 98.8) | 6808 | 10,020,000 | 1.4 (1.0, 1.7) | 103 | 130,000 | 0.2 (0.1, 0.3) | 18 | 10,000 |
| **2016** | 98.8 (98.5, 99.2) | 8044 | 10,040,000 | 1.0 (0.7, 1.3) | 79 | 100,000 | —^§§^ | — | — |
| **2017** | 98.9 (98.5, 99.2) | 6490 | 10,410,000 | 1.1 (0.7, 1.4) | 65 | 110,000 | — | — | — |
| **2018** | 97.7* (97.2, 98.2) | 7692 | 10,150,000 | 1.8* (1.4, 2.2) | 148 | 180,000 | 0.4* (0.2, 0.7) | 37 | 40,000 |
| **2019^^^** | 94.7 (93.9, 95.6) | 7247 | 9,750,000 | 4.2 (3.5, 5.0) | 317 | 430,000 | 1.0 (0.7, 1.4) | 87 | 100,000 |
| **Former combustible user** | | | | | | | | | |
| **2014** | 89.5 (85.7, 93.3) | 921 | 1,060,000 | 8.2 (5.1, 11.3) | 101 | 90,000 | 2.2 (0.9, 3.6) | 33 | 20,000 |
| **2015** | 85.1 (81.8, 88.4) | 631 | 950,000 | 11.3 (8.2, 14.3) | 88 | 120,000 | 3.6 (2.2, 5.1) | 33 | 40,000 |
| **2016** | 86.9 (83.6, 90.2) | 829 | 960,000 | 9.1 (6.1, 12.2) | 78 | 100,000 | 4.0 (2.2, 5.7) | 29 | 40,000 |
| **2017** | 90.6 (86.8, 94.3) | 534 | 740,000 | 6.6 (3.9, 9.3) | 42 | 50,000 | — | — | — |
| **2018** | 84.0* (80.5, 87.6) | 588 | 720,000 | 10.8* (7.7, 13.9) | 82 | 90,000 | 5.2 (2.9, 7.4) | 39 | 40,000 |
| **2019^^^** | 66.7 (61.3, 72.1) | 508 | 640,000 | 20.8 (16.6, 25.1) | 152 | 200,000 | 12.4 (9.1, 15.8) | 92 | 120,000 |
| **Current combustible user** | | | | | | | | | |
| **2014** | 60.3 (51.0, 69.7) | 301 | 330,000 | 23.9 (17.8, 30.1) | 153 | 130,000 | 15.7 (11.0, 20.5) | 90 | 80,000 |
| **2015** | 36.1* (28.3, 43.9) | 130 | 160,000 | 35.8* (29.0, 42.5) | 125 | 160,000 | 28.1* (22.9, 33.3) | 102 | 120,000 |
| **2016** | 51.7* (45.0, 58.4) | 202 | 250,000 | 26.6* (21.4, 31.9) | 101 | 130,000 | 21.7 (16.3, 27.1) | 90 | 100,000 |
| **2017** | 50.1 (40.6, 59.6) | 163 | 190,000 | 26.9 (17.5, 36.2) | 74 | 100,000 | 23.0 (16.5, 29.5) | 66 | 80,000 |
| **2018** | 46.2 (38.3, 54.1) | 154 | 160,000 | 22.9 (17.5, 28.3) | 71 | 80,000 | 30.9 (24.3, 37.6) | 77 | 110,000 |
| **2019^^^** | 30.8 (24.9, 36.7) | 139 | 160,000 | 32.1 (27.4, 36.9) | 121 | 170,000 | 37.1 (29.5, 44.6) | 135 | 200,000 |

Numbers in parentheses represent 95% confidence intervals.

Wtd = weighted. Estimated total number of users was rounded down to the nearest 10,000 persons.

*p-value <0.05 for the difference in prevalence compared to the previous year in a general linear model simultaneously testing the effect of smoking status, year, and the interaction between smoking status and year.

Past 30-day use of e-cigarettes was determined by asking, “During the past 30 days, on how many days did you use e-cigarettes?” Combustible tobacco includes cigarettes (including roll-your-own), cigars (cigars, cigarillos, little cigars), pipe tobacco, hookah, or bidis. Never combustible users report never using any combustible tobacco products, not even “one or two puffs”. Former combustible users report ever using any combustible tobacco products, even “one or two puffs”, but no use in the past 30 days. Current combustible users report using any combustible tobacco product in the past 30 days.

^§§^Dashes indicate estimates that are unreliable because the relative standard error was >30% or the unweighted denominator was < 50.

^a^Infrequent = used e-cigarettes 1-5 days in the past 30 days; Moderate or Frequent = 6-30 days.

^^^Data from 2019 are not directly comparable with estimates in previous years due to changes in the NYTS format from paper to electronic.
